# Role of uric acid as a biomarker of cognitive function in schizophrenia during maintenance period

**DOI:** 10.3389/fpsyt.2023.1123127

**Published:** 2023-03-22

**Authors:** Zelin Yuan, Huamin Liu, Xiaochun Zhang, Yong He, Shanyuan Gu, Dan Mo, Shaoli Wang, Zhiwei Huang, Keyi Wu, Rui Zhou, Qi Zhong, Yining Huang, Bifei Cao, Haowen Chen, Xianbo Wu

**Affiliations:** ^1^Department of Epidemiology, School of Public Health, Southern Medical University (Guangdong Provincial Key Laboratory of Tropical Diseases), Guangzhou, China; ^2^Department of Psychiatry, Baiyun Jingkang Hospital, Guangzhou, Guangdong, China

**Keywords:** schizophrenia, cognitive function, uric acid, antioxidant, biomarker

## Abstract

**Background:**

Previous studies involving uric acid (UA) in some specialized disease populations have found that high UA is associated with enhanced patient function. The mechanism to explain this association may be that UA, an important antioxidant, exerts neuroprotective effects. Patients with schizophrenia (SCZ) have severe oxidative stress abnormalities, and cognitive impairment is a major obstacle to their rehabilitation. Only few studies have been conducted on UA and cognitive impairment in SCZ. This study aims to clarify the relationship between UA and cognitive impairment and explore whether UA could be used as a potential biological marker of cognition in SCZ during maintenance period.

**Methods:**

A total of 752 cases of SCZ during maintenance period from Baiyun Jingkang Hospital were included. Cognition was measured using the Mini-Mental State Examination scale. UA was measured using the Plus method. The participants were grouped on the basis of UA to evaluate the association of cognition with low-normal (3.50–5.07 mg/dL for men, 2.50–4.19 mg/dL for women), middle-normal (5.07–6.39 mg/dL for men, 4.19–5.18 mg/dL for women), high-normal (6.39–7.00 mg/dL for men, 5.18–6.00 mg/dL for women), and high (>7.00 mg/dL for men, >6.00 mg/dL for women) levels of UA. Multiple logistic regression and linear regression models and restricted cubic spline (RCS) were utilized to evaluate the relationship.

**Results:**

Uric acid was positively associated with cognitive function. Subgroup analyses showed that high UA was associated with enhanced cognition in participants with low anticholinergic cognitive burden (ACB).

**Conclusion:**

Uric acid may be used as a simple objective biological indicator to assess cognition in SCZ during maintenance period.

## 1. Introduction

For decades, some researchers regarded UA as a risk factor. UA can cause gout attacks, and it is a risk factor that promotes the occurrence and progression of many diseases, such as chronic kidney disease (1), metabolic syndrome, and cardiovascular disease (2, 3). However, UA is not useless; it has important physiological roles. UA has a similar structure to brain stimulants, such as caffeine and theobromine, and it has been linked to improved intelligence (4, 5). UA was also found to be effective in reducing inflammation levels (6). A notable detail that many studies have demonstrated the powerful antioxidant properties of UA. Of the total antioxidant capacity of human plasma, the antioxidant capacity of UA alone accounts for approximately 50%, which is almost equal to that of ascorbic acid (7, 8). UA could also scavenge nitrogen peroxide radicals (9), trap free radicals in the peroxynitrite anion cascade (10), and inhibit the iron-mediated oxidation of ascorbic acid in human blood (11, 12). Therefore, UA could indicate the oxidative stress state of the body. In previous studies on healthy adults, UA has been shown to be neuroprotective and reduce the risk of neurodegenerative diseases; low UA has also been found to be a risk factor for cognitive impairment (13–16), with oxidative stress being an important reason for this effect (7). Similar results have been revealed in other disease populations, such as high vascular burden population (17), post-stroke patients (18), patients with Parkinson’s disease (19), and patients with depression (20).

Schizophrenia (SCZ), one of the most serious mental diseases, severely affects the function and quality of life of patients (21). The lifetime prevalence of this disease is approximately 1% (22). Cognitive impairment, as one of the three core symptoms of SCZ, is present in up to 80% of patients with SCZ (23). It could affect the treatment effect and prognosis of patients with SCZ and increase the risk of disability. Although antipsychotics are effective in the treatment of positive symptoms in patients with SCZ, they have little effect on improving negative symptoms and cognitive deficits (24, 25). Although the pathogenesis of SCZ remains unclear, oxidative stress has been increasingly implicated in the pathophysiology of SCZ (26, 27). Patients with SCZ showed increased lipid peroxide levels in the blood, altered levels of enzymatic or non-enzymatic antioxidants (28), and abnormal manifestations of oxidative stress in the body (10, 29). Meta-analysis results suggested that oxidative stress is elevated in patients with SCZ in maintenance period (30). Case-control studies have been conducted on the relationship between SCZ and UA, but their results are controversial, possibly due to the limitation of sample size. Although the SCZ group had higher UA levels than normal participants, opposite results were observed in other studies (20, 31). These case controls exist only to compare the UA values between patients and healthy subjects, and they did not involve the risk factors of SCZ. The study of potential risk factors of SCZ should be more limited to this subgroup of SCZ population. Notably, these previous studies did not address cognitive deficits, which are the main barriers to recovery in patients with SCZ. Moreover, the relationship between cognitive function in maintenance SCZ and UA, an important potential neuroprotective factor, remains unclear. Therefore, in the present study, the association between cognitive function and UA in SCZ was explored.

This study aimed to explore the possibility of UA as a biomarker of cognitive dysfunction in SCZ during maintenance period and whether oxidative stress in SCZ is one of the potential physiological mechanisms of cognitive dysfunction by studying the relationship between cognitive dysfunction and UA in SCZ during maintenance period to provide possible treatment ideas.

## 2. Materials and methods

### 2.1. Study population

The medical records and blood biochemical information of 916 patients with SCZ receiving maintenance treatment in Guangzhou Baiyun Jingkang Hospital, Guangdong Province, from 2020 to 2021 were collected. A total of 752 patients with confirmed SCZ during maintenance period were included in this study. A total of 164 patients with depression, bipolar disorder, organic brain disease, and non-cooperation; without cognitive testing and UA indicators; and using UA-lowering medications were excluded. All participants received standardized treatment during the maintenance period of SCZ, and their lifestyle was managed uniformly by the hospital. All participants signed an informed consent form. This study was approved by the Ethics Committee of the study hospital (NFYKDX002).

### 2.2. Cognitive assessment

The Chinese version of Mini-Mental State Examination (MMSE) scale was used to assess the cognitive function of the patients by professionally trained investigators. For each of the 30 questions, a score of 1 was awarded for correct answers and a score of 0 was awarded for incorrect or unknown answers, with high scores indicating good cognitive function. This scale has been shown to have good reliability and validity in patients with SCZ (32). Cognitive impairment was defined by educational background combined with MMSE score (33): illiteracy with MMSE ≤ 16, primary school with MMSE ≤ 19, secondary school education or above with MMSE ≤ 23 were defined as cognitive impairment. Most of the MMSE items were from the literal translation of the original manuscript, and in order to meet the needs of Chinese cultural background, some items had been adapted (34). Chinese guidelines for the diagnosis and treatment of dementia and cognitive impairment have adopted the Chinese version of MMSE as one of the main assessment methods of clinical cognitive function (35).

### 2.3. Measurements of UA

Uniformly trained staff following standard protocols collected blood samples from the participants after overnight fasting. The UA level (mg/dL) was analyzed using the UA Plus method. On the basis of UA levels, the participants were divided into the following groups: low-normal (3.50–5.07 mg/dL for men and 2.50–4.19 mg/dL for women), middle-normal (5.07–6.39 mg/dL for men and 4.19–5.18 mg/dL for women), high-normal (6.39–7.00 mg/dL for men and 5.18–6.00 mg/dL for women), and high (>7.00 mg/dl for men and >6.00 mg/dl for women) groups.

### 2.4. Covariates

The patients’ age, gender, SCZ course, educational level, smoking history, drinking history, fasting plasma glucose (FPG), triglyceride (TG), total cholesterol (TC), low-density lipoprotein cholesterol (LDLC), high-density lipoprotein cholesterol (HDLC), urea, albumin, creatinine (CRE), Brief Psychiatric Rating Scale (BPRS) score, and current prescription drugs were extracted from medical records and biochemical test reports. Educational level was divided into primary school and below, junior high school, senior high school, and above. Body mass index (BMI) was calculated as weight (kg) divided by height (m) squared. Hypertension was defined as clinician diagnosis, systolic blood pressure ≥ 140 mmHg, or diastolic blood pressure ≥ 90 mmHg. Diabetes was defined as clinician’s diagnosis, fasting blood glucose ≥ 126 mg/dL, or glycosylated hemoglobin ≥ 6.5%. Dyslipidemia was defined as one or more of TG ≥ 2.26 mmol/L, LDLC ≥ 4.14 mmol/L, HDLC < 1.04 mmol/L, and TC ≥ 6.22 mmol/L or current use of lipid-improving drugs. The ACB scale was used to assess participants’ ACB. The ACB scale is an expert-validated scale for assessing anticholinergic properties of drugs (36, 37). The ACB scale assigns a dose-independent rating to each drug according to its anticholinergic properties: low activity = 1; moderate activity = 2; strong activity = 3. The ACB values and frequency distributions for each drug are provided in Supplementary Table 2. The ACB score for each patient was calculated as the sum of the ACB score for each antipsychotic drug and the ACB score for the combination drug for that patient (38), and the first-generation and second-generation antipsychotics, benzodiazepines, hypnotics, sedatives, and other drugs, among others, were considered in the ACB score of this study. According to the five subscales of BPRS proposed by Shafer (39), the 18 items of BPRS are divided into five categories: affect, positive symptoms, negative symptoms, resistance and activation. The Chinese version of BPRS has been confirmed to have good validity and reliability (40, 41).

### 2.5. Statistical analysis

Continuous variables that were not normally distributed or had heterogeneity of variance were presented as median [interquartile range]. The remaining continuous variables were presented as means (standard deviations). Categorical variables were presented as percentages. Depending on the situation, Kruskal–Wallis test or analysis of variance was used for continuous variables. Chi-square test was used for classified variables. Logistic regression and linear regression models were used to examine the association between UA levels and cognitive impairment in patients with SCZ. Three models with different adjustment factors were fitted. Next, RCS nodes based on UA quartiles were used to visualize the association between UA levels and cognitive impairment in patients with SCZ.

Subgroup analyses were performed to examine whether the association between UA and cognitive impairment in patients with SCZ was altered by ACB scores in the multivariate Logistic regression and linear regression models. All data were analyzed by using STATA version 17 (StataCorp LP, College Station, TX, USA).

## 3. Results

### 3.1. Baseline characteristics

The baseline characteristics of the study population are listed in Table 1. The low-normal, middle-normal, high-normal, and high groups had 206, 206, 206, and 134 participants, respectively. The average age of the total population was 48.9 ± 12.2 years, and males accounted for 54.79% of the total population. Those with higher UA levels were more likely to have smoking and drinking histories; non-cognitive impairment; dyslipidemia; higher BMI, TG, TC, LDLC, and CRE; and lower HDLC (Table 1). Statistically significant differences also were found in the use of risperidone and aripiprazole (*P* < 0.05).

**TABLE 1:** Baseline characteristics.

	All participants (n = 752)	Low-normal (n = 206)	Middle-normal (n = 206)	High-normal (n = 206)	High (n = 134)	*P*-value
Age, years ± SD	48.9 ± 12.2	49.4 ± 11.7	50.7 ± 12.7	47.5 ± 12.1	47.5 ± 11.8	0.691
Male (n, %)	412 (54.79)	109 (52.91)	109 (52.91)	109 (52.91)	85 (63.43)	0.178
BMI, kg/m^2^	22.77 (20.20–25.30)	22.07 (19.47–24.38)	22.77 (20.61–24.53)	22.86 (20.45–25.79)	23.95 (20.94–25.91)	**<0.001**
Course	16 (8–25)	17 (7–24)	15 (8–26)	16 (9–25)	16 (10–25.5)	0.967
UA, mg/dL	5.45 ± 1.57	3.87 ± 0.59	4.92 ± 0.48	5.93 ± 0.61	7.94 ± 1.26	**<0.001**
Educational level						0.115
Primary school or lower	221 (29.39)	51 (24.76)	69 (33.50)	52 (25.24)	49 (36.57)	
Middle school	330 (43.88)	100 (48.54)	88 (42.72)	92 (44.66)	50 (37.31)	
High school or above	201 (26.73)	55 (26.70)	49 (23.79)	62 (30.10)	35 (26.12)	
Smoking history	87 (11.57)	16 (7.77)	15 (7.28)	25 (12.14)	31 (23.13)	**<0.001**
Drinking history	59 (7.85)	14 (6.80)	11 (5.34)	16 (7.77)	18 (13.43)	**0.048**
Cognitive impairment	510 (67.82)	151 (73.30)	152 (73.79)	130 (63.11)	77 (57.46)	**0.003**
Diabetes	74 (9.84)	13 (6.31)	23 (11.17)	22 (10.68)	16 (11.94)	0.248
Hypertension	261 (34.71)	68 (33.01)	78 (37.86)	66 (32.04)	49 (36.57)	0.568
Dyslipidemia	510 (67.82)	110 (53.40)	127 (61.65)	159 (77.18)	114 (85.07)	**<0.001**
TG, mmol/L	1.10 (1.00–2.00)	1.00 (1.00–1.40)	1.10 (1.00–2.00)	1.10 (1.00–2.00)	2.00 (1.10–2.80)	**<0.001**
TC, mmol/L	4.60 (4.00–5.00)	4.50 (4.00–5.00)	4.45 (4.00–5.00)	4.60 (4.00–5.00)	5.00 (4.00–5.30)	**0.028**
LDLC, mmol/L	2.72 (2.00–3.00)	2.37 (2.00–3.00)	2.52 (2.00–3.00)	2.91 (2.20–3.00)	3.00 (2.34–3.32)	**<0.001**
HDLC, mmol/L	1.00 (0.99–1.16)	1.04 (1.00–1.38)	1.00 (1.00–1.20)	1.00 (0.94–1.07)	1.00 (0.86–1.00)	**<0.001**
Urea, mmol/L	3.00 (2.60–4.00)	3.00 (2.70–4.00)	3.30 (2.80–4.00)	3.00 (2.50–4.00)	3.00 (2.40–3.90)	0.074
Albumin, g/L	43 (41–48)	43 (41–48)	43 (40–48)	43 (40–46)	44 (41–48)	0.284
FPG, mmol/L	4.84 (4.18–5.00)	4.83 (4.33–5.00)	4.84 (4.26–5.00)	4.83 (4.12–5.00)	4.99 (4.03–5.18)	0.974
CRE, mg/dL	68.88 ± 17.85	62.79 ± 15.85	66.55 ± 15.96	71.63 ± 17.89	77.35 ± 19.23	**0.032**
Clozapine	165 (21.94)	50 (24.27)	43 (20.87)	44 (21.36)	28 (20.90)	0.821
Olanzapine	264 (35.11)	70 (33.98)	86 (41.75)	69 (33.50)	39 (29.10)	0.091
Quetiapine	91 (12.10)	28 (13.59)	26 (12.62)	26 (12.62)	11 (8.21)	0.486
Risperidone	320 (42.55)	103 (50.00)	70 (33.98)	88 (42.72)	59 (44.03)	**0.012**
Aripiprazole	67 (8.91)	16 (7.77)	18 (8.74)	29 (14.08)	4 (2.99)	**0.005**
Sodium valproate	176 (3.38)	47 (22.82)	47 (22.82)	44 (21.36)	38 (28.36)	0.495
Chlorpromazine	47 (6.25)	13 (6.31)	11 (5.34)	11 (5.34)	12 (8.96)	0.521
Perphenazine	72 (9.57)	12 (5.83)	21 (10.19)	22 (10.68)	17 (12.69)	0.156
ACB	3 (1–4)	3 (1–4)	3 (1–4)	3 (1–4)	3 (1–4)	0.528
MMSE	18.32 ± 7.24	17.90 ± 6.80	17.64 ± 6.84	18.63 ± 7.80	19.56 ± 7.50	0.078
Orientation	6.32 ± 2.78	5.79 ± 2.63	6.19 ± 2.71	6.37 ± 2.91	7.05 ± 2.71	**0.004**
Immediate memory	1.91 ± 1.27	1.67 ± 1.21	1.75 ± 1.28	2.11 ± 1.32	2.11 ± 1.17	**0.003**
Attention and numeracy	2.24 ± 1.85	1.96 ± 1.74	2.16 ± 1.77	2.26 ± 1.87	2.63 ± 2.01	**0.040**
Delayed recall	1.41 ± 1.16	1.21 ± 1.16	1.28 ± 1.16	1.48 ± 1.12	1.71 ± 1.17	**0.003**
Languages	5.12 ± 2.34	4.60 ± 2.09	4.85 ± 2.35	5.27 ± 2.48	5.85 ± 2.23	**<0.001**
BPRS	43.76 ± 9.28	44.13 ± 11.14	44.81 ± 10.17	43.14 ± 7.95	42.65 ± 6.12	0.670
Affect	9.25 ± 2.85	9.17 ± 3.46	9.29 ± 2.80	9.28 ± 2.64	9.27 ± 2.15	0.973
Positive symptoms	9.73 ± 3.46	9.98 ± 3.66	9.52 ± 3.54	9.92 ± 3.55	9.41 ± 2.77	0.324
Negative symptoms	10.37 ± 3.55	10.25 ± 3.71	10.41 ± 3.50	10.61 ± 3.66	10.13 ± 3.16	0.623
Resistance	8.25 ± 3.16	8.37 ± 3.73	8.23 ± 3.04	8.28 ± 3.13	8.06 ± 2.33	0.865
Activation	6.21 ± 2.60	6.26 ± 3.63	6.11 ± 2.17	6.25 ± 2.12	6.26 ± 1.88	0.931

BMI, body mass index; UA, uric acid; TG, triglyceride; TC, total cholesterol; LDLC, low-density lipoprotein cholesterol; HDLC, high-density lipoprotein cholesterol; FPG, fasting plasma glucose; CRE, creatinine; ACB, Anticholinergic Cognitive Burden; MMSE, Mini-Mental State Examination; BPRS, Brief Psychiatric Rating Scale. *P*-values < 0.05 were highlighted in bold.

### 3.2. Association between UA levels and cognitive function

**FIGURE 1:**
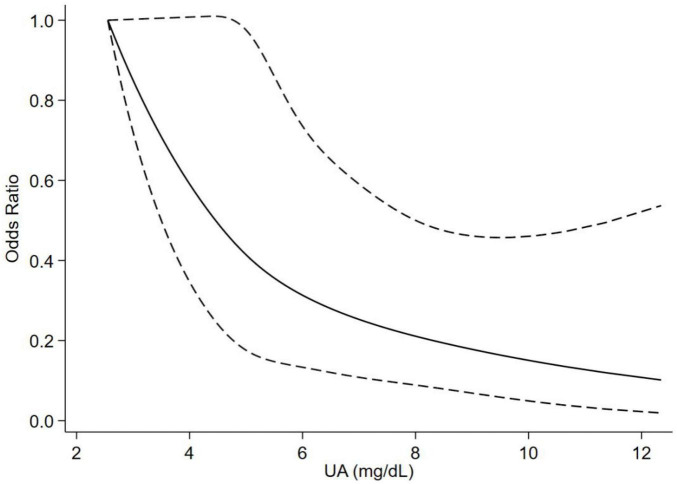
Association between UA levels and cognitive impairment in the SCZ population based on the restricted cubic spline model. Adjusted for age, sex, BMI, course, smoking history, drinking history, hypertension, diabetes, dyslipidemia, creatinine, risperidone, aripiprazole, and BPRS. UA, uric acid; SCZ, schizophrenia; BMI, body mass index; BPRS, Brief Psychiatric Rating Scale.

After adjusting for age, sex, BMI, course, smoking history, drinking history, hypertension, diabetes, dyslipidemia, CRE, risperidone, aripiprazole, and BPRS, the participants with higher UA levels had an incrementally better cognition (*P*_for trend_ < 0.001) in model 3. The ORs (95% CI) of the participants were 0.530 (0.324, 0.869) for the high-normal group, and 0.396 (0.224, 0.701) for the high group relative to that for the low-normal group (Table 2). In addition, the OR (95% CI) of UA (for each unit standard deviation increase) associated with cognitive impairment in total participants was 0.564 (0.445, 0.714) in model 3 (Table 3). The results of models 1 and 2 were similar to those of model 3 (Tables 2, 3). Then, the relationship between UA levels and different cognitive domains was analyzed. After adjusting for the same covariates as above, UA level was positively correlated with cognitive scores (global cognition, orientation, delayed recall, and languages). The β values (95% CI) of the participants were 0.595 (0.170, 1.020) in global cognition, 0.159 (0.002, 0.315) in orientation, 0.092 (0.027, 0.157) in delayed recall, and 0.225 (0.100, 0.349) in languages in model 3 (Table 4). The RCS results revealed no non-linear relationship between UA levels and cognitive impairment in patients with SCZ during maintenance period (Figure 1).

**TABLE 2:** Association between UA and cognitive function.

UA	Cognitive impairment n (%)	Model 1	Model 2	Model 3
Low-normal	151 (73.30)	Ref.	Ref.	Ref.
Middle-normal	152 (73.79)	1.168 (0.721, 1.891)	1.095 (0.673, 1.782)	1.051 (0.634, 1.742)
High-normal	130 (63.11)	0.655 (0.415, 1.035)	**0.570 (0.356, 0.913)**	**0.530 (0.324, 0.869)**
High	77 (57.46)	**0.549 (0.328, 0.919)**	**0.444 (0.259, 0.762)**	**0.396 (0.224, 0.701)**
*P* for trend		**0.004**	**<0.001**	**<0.001**

Model 1: Adjusted for age, sex, BMI, course, smoking history, and drinking history. Model 2: Adjusted for age, sex, BMI, course, smoking history, drinking history, hypertension, diabetes, and dyslipidemia. Model 3: Adjusted for age, sex, BMI, course, smoking history, drinking history, hypertension, diabetes, dyslipidemia, creatinine, risperidone, aripiprazole, and BPRS. UA, uric acid; BMI, body mass index; BPRS, Brief Psychiatric Rating Scale. ORs 95% CI without 1, or *P*-values < 0.05 were highlighted in bold.

**TABLE 3:** Association between UA (for each unit standard deviation increase) and cognitive impairment.

	Cognitive impairment n (%)	Model 1	Model 2	Model 3
**Total participant**	615 (81.78)			
UA		**0.637 (0.517, 0.785)**	**0.552 (0.440, 0.692)**	**0.564 (0.445, 0.714)**
**Subgroup analysis**				
Low ACB (0–3)	292 (77.66)			
UA		**0.466 (0.342, 0.636)**	**0.380 (0.267, 0.539)**	**0.371 (0.254, 0.544)**
High ACB (3–13)	323 (85.90)			
UA		0.836 (0.587, 1.192)	0.737 (0.504, 1.077)	0.790 (0.525, 1.190)
*P* for interaction		**0.009**	**0.009**	**0.008**

Model 1: Adjusted for age, sex, BMI, course, smoking history, and drinking history. Model 2: Adjusted for age, sex, BMI, course, smoking history, drinking history, hypertension, diabetes, and dyslipidemia. Model 3: Adjusted for age, sex, BMI, course, smoking history, drinking history, hypertension, diabetes, dyslipidemia, creatinine, risperidone, aripiprazole, and BPRS. UA, uric acid; BMI, body mass index; BPRS, Brief Psychiatric Rating Scale. ORs 95% CI without 1, or *P*-values <0.05 were highlighted in bold.

**TABLE 4:** Association between UA and cognitive function.

	UA (mg/dL)		
	**Model 1**	**Model 2**	**Model 3**
Global cognition	**0.423 (0.030, 0.817)**	**0.663 (0.258, 1.069)**	**0.595 (0.170, 1.020)**
Orientation	0.118 (−0.026, 0.261)	**0.182 (0.033, 0.331)**	**0.159 (0.002, 0.315)**
Immediate memory	0.042 (−0.024, 0.107)	**0.080 (0.013, 0.148)**	0.071 (−0.001, 0.1430
Attention and numeracy	0.014 (−0.083, 0.110)	0.051 (−0.049, 0.152)	0.044 (−0.061, 0.150)
Delayed recall	**0.072 (0.012, 0.133)**	**0.105 (0.043, 0.167)**	**0.092 (0.027, 0.157)**
Languages	**0.174 (0.060, 0.288)**	**0.241 (0.124, 0.359)**	**0.225 (0.100, 0.349)**

Model 1: Adjusted for age, sex, BMI, course, smoking history, and drinking history. Model 2: Adjusted for age, sex, BMI, course, smoking history, drinking history, hypertension, diabetes, and dyslipidemia. Model 3: Adjusted for age, sex, BMI, course, smoking history, drinking history, hypertension, diabetes, dyslipidemia, creatinine, risperidone, aripiprazole, and BPRS. MMSE, Mini-Mental State Examination; UA, uric acid; BMI, body mass index; BPRS, Brief Psychiatric Rating Scale. βs 95% CI without 0 were highlighted in bold.

### 3.3. Association between UA levels and cognitive function stratified by ACB

After stratification by the median ACB and adjusting for additional confounders, the association of UA (for each unit standard deviation increase) with cognitive impairment was found to be significant (Table 3). In the low ACB subgroup, high UA levels were associated with good cognitive function (OR = 0.371, 95% CI: 0.254–0.544) in model 3. The other two models with different adjustments also showed significance, and their interaction (*P*_for interaction_ < 0.05) were observed. Stratified analysis of linear regression showed that UA levels were positively associated with cognitive scores in participants with low ACB. The β values (95% CI) of the participants were 1.008 (0.403, 1.613) in global cognition, 0.308 (0.087, 0.528) in orientation, 0.129 (0.024, 0.234) in immediate memory, 0.156 (0.065, 0.247) in delayed recall, and 0.372 (0.187, 0.558) in languages in model 3 (Table 5).

**TABLE 5:** Association between UA and cognitive function (stratified by the median ACB).

	UA (mg/dL)		
	**Model 1**	**Model 2**	**Model 3**
**Low ACB (0–3)**			
Global cognition	**0.854 (0.291, 1.418)**	**1.088 (0.519, 1.656)**	**1.008 (0.403, 1.613)**
Orientation	**0.249 (0.046, 0.453)**	**0.323 (0.117, 0.529)**	**0.308 (0.087, 0.528)**
Immediate memory	**0.110 (0.016, 0.204)**	**0.138 (0.042, 0.234)**	**0.129 (0.024, 0.234)**
Attention and numeracy	0.012 (−0.125, 0.149)	0.054 (−0.086, 0.193)	0.043 (−0.106, 0.192)
Delayed recall	**0.144 (0.061, 0.227)**	**0.170 (0.085, 0.254)**	**0.156 (0.065, 0.247)**
Languages	**0.339 (0.167, 0.510)**	**0.403 (0.229, 0.577)**	**0.372 (0.187, 0.558)**
**High ACB (3–13)**			
Global cognition	−0.045 (−0.586, 0.495)	0.111 (−0.466, 0.687)	0.099 (−0.519, 0.718)
Orientation	−0.014 (−0.218, 0.190)	0.007 (−0.212, 0.225)	0.002 (−0.233, 0.237)
Immediate memory	−0.042 (−0.134, 0.051)	0.001 (−0.097, 0.098)	−0.016 (−0.121, 0.090)
Attention and numeracy	0.015 (−0.122, 0.152)	0.031 (−0.116, 0.178)	0.033 (−0.123, 0.190)
Delayed recall	−0.019 (−0.105, 0.067)	0.011 (−0.081, 0.104)	−0.004 (−0.102, 0.093)
Languages	0.006 (−0.142, 0.154)	0.051 (−0.106, 0.208)	0.073 (−0.097, 0.244)

Model 1: Adjusted for age, sex, BMI, course, smoking history, and drinking history. Model 2: Adjusted for age, sex, BMI, course, smoking history, drinking history, hypertension, diabetes, and dyslipidemia. Model 3: Adjusted for age, sex, BMI, course, smoking history, drinking history, hypertension, diabetes, dyslipidemia, creatinine, risperidone, aripiprazole, and BPRS. MMSE, Mini-Mental State Examination; UA, uric acid; BMI, body mass index; BPRS, Brief Psychiatric Rating Scale. βs 95% CI without 0 were highlighted in bold.

## 4. Discussion

This study provided additional evidence showing that high serum UA levels are associated with good cognitive status in patients with SCZ during maintenance period. In addition, the stratified results suggested that the association between UA and cognitive function varied among participants on the basis of ACB and was strong among those with low ACB. This association remained significant after adjusting for a wide array of health-related variables.

This study demonstrated the association between high serum UA levels and good cognitive status (orientation, immediate memory, delayed recall, and languages) in patients with SCZ during maintenance period. A meta-analysis reported similar conclusions regarding non-SCZ population (42). A growing body of evidence suggested that in degenerative neuropathy, UA exerts neuroprotective properties by inhibiting inflammation and *via* antioxidation effects (43, 44). Studies using mouse experimental models found that exogenous UA treatment could inhibit inflammation and oxidative stress in the central nervous system (45). Therefore, the association between UA and cognitive function in SCZ may be partially explained by oxidative stress. Serum UA could reflect the total antioxidant capacity of serum (46, 47). The reduction in UA levels has been previously confirmed to be a marker of abnormal antioxidant defense systems in patients with SCZ (48). Indeed, this was the case given that UA levels are significantly lower in patients with SCZ than in healthy controls (31, 49, 50).

Uric acid levels have been associated with negative symptoms after treatment in patients with SCZ in the acute phase (51) and negatively associated with symptom severity (52). Studies on UA and cognitive impairment (one of the three major symptoms of SCZ and the main barrier to SCZ rehabilitation) in patients with SCZ have only been conducted on first-episode, drug-free populations and found no statistically significant associations (53). The reality is that most patients with SCZ are in maintenance treatment period, and studies on populations with maintenance SCZ have deeper clinical guiding significance than those on first-episode untreated populations. However, the relationship between UA and cognitive impairment in patients with SCZ in maintenance period, an important period, remains largely unexplored. A genome-wide association study suggested that low UA may be associated with SCZ pathogenesis and is more suitable for diagnostic and therapeutic testing than other markers (54). The findings of the present study suggested that high serum UA may serve as a potential blood biomarker for the assessment of cognitive improvement in SCZ. Additional evidence was also provided, showing that high levels of UA within the normal range are associated with good cognitive functional status and serum UA levels above the normal reference range are associated with high cognitive function. In addition to these, we found that UA levels were negatively correlated with the severity of positive symptoms in SCZ patients (Supplementary Table 1). Long-term cohort studies must be established and optimal serum UA levels must be determined to balance the combined consideration of gout, cardiovascular disease and cognitive impairment, and positive and negative symptoms.

The high utilization rate of oxygen in the brains of people with mental illness, the increase in the number of produce free radicals, and antioxidant defense mechanism, and excitability toxicity resulted in a particular susceptibility to oxidative stress injury (55). Although the pathological mechanism of SCZ is not fully understood, mitochondrial function and redox imbalance have been shown to be key factors in the occurrence and progression of SCZ (56). Furthermore, a mechanistic review of the pathological process of SCZ mentioned that high levels of nitric oxide and peroxynitrite in SCZ patients produce cytotoxic effects on oligodendrocytes (57), nitrite peroxide as a wide range of bioactive molecules strong oxidizer, nitric oxide and oxygen nitrite interactions, the formation of hydrogen peroxide, thus inhibiting mitochondrial respiration (58). UA, as an inhibitor of specific peroxynitrite mediated reaction (59), may protect mitochondrial function and delay the progression of SCZ through this mechanism. Previous studies have confirmed that treatment can improve the level of UA to have oxygen nitrite-mediated diseases of the nervous system (60). In addition, UA has also been found to have protective effects during inflammatory processes in the central nervous system. Exogenous UA treatment protects the integrity of the blood brain barrier and can reduce the permeability of the blood brain barrier to inflammatory cells (45). The anti-inflammatory effect was also found to be UA dose-dependent in rat experiments (6). The pathological mechanisms involved in these studies may support our conclusion that higher UA is associated with better cognition in SCZ patients.

Another important finding of this study is the better association of UA with cognitive function in the low ACB group, wherein ACB interacted with UA, than in other groups. In older adults, an ACB score of 3 is associated with a 50% increase in the risk of cognitive impairment (61). In the present study, the patients with SCZ in maintenance period had a high anticholinergic burden, with an average ACB score of 3, and 65.03% of the participants had ACB ≥ 3. Therefore, subgroup analysis was performed in accordance with the level of ACB. Previous studies have found that although high-ACB antipsychotic drugs may be important for alleviating symptoms and maintaining normal function, their long-term use may impair cognitive function (62). Reducing the use of high-ACB antipsychotic drugs could improve cognitive function and quality of life (63). Therefore, the choice of drugs and the personalization of drug combination according to cognitive status could be important.

In addition, the high UA group had the highest mean BMI [23.95 (20.94–25.91)] and MMSE, this seems to imply some association between BMI and cognitive function. In fact, the mean BMI in the high UA group was still less than the critical reference value of 24 kg/m^2^ for overweight. Studies involving older community populations have found that overweight but not obese individuals have a lower risk of cognitive impairment than those with stable weight; obese people gaining weight will increase the risk of cognitive impairment (64). One possible reason for this is that some hormones with neuroprotective effects are highly expressed in people with high BMI, such as estrogen secretion in the extracranial glandular tissue of overweight women (65) and leptin secretion in the adipose tissue of people with a high BMI (66, 67). And the possible mechanism of skeletal reduce disease accompanied by BMI lower, which could lead to a low level of physical activity and an eventual decline in cognitive ability (68). Given the inadequate sample of obese participants in this study, the findings should be interpreted with caution. Future studies will be conducted in this regard.

The relationship between brain and plasma levels of UA was one of the foundations of this study: serum UA levels were 10 times higher in healthy participants than in their cerebrospinal fluid (CSF) (69). CSF UA may be an indicator of brain cell damage in participants with neurodegenerative diseases (70, 71). In fact, the permeability of the blood-brain barrier is increased in the state of neurodegenerative diseases. Part of the CSF UA is still converted by nucleic acid from injured brain cells, and part of the plasma UA passes through the blood-brain barrier into the CSF. Therefore, CSF UA is determined by both plasma UA and the integrity of the blood-brain barrier (72). Case-control studies on cognitive impairment and CSF UA have also yielded inconsistent conclusions when comparing CSF UA levels between control and case groups (73, 74). Thus, the ratio of UA in blood to UA in CSF may be an indicator worth exploring. The relationship between peripheral blood and central UA metabolism needs further experimental proof.

It was found that other plasma antioxidants such as vitamin C, albumin, and bilirubin were also significantly decreased in SCZ patients (48, 75). Among them, vitamin C can be used as an adjuvant treatment for antioxidant in neuropsychiatric disorders (76). The results of this study and previous works suggest the possibility of UA as a biomarker for a variety of neurodegenerative diseases. It will be worthwhile to find more specific and sensitive biomarkers for the metabolic process and cognitive decline in SCZ patients.

This study has several strengths. First, the current assessment of cognitive function in hospitalized patients with SCZ mostly used questionnaires. Such an assessment approach takes a long time and requires trained professional investigators. This study provided a simple and easy-to-measure index for assessing the cognitive status of patients with SCZ during maintenance period, and this index could be used to screen people at high risk of cognitive decline. Second, this study included data on many possible confounders with reasonable adjustment. The patients’ lifestyles were managed by the hospital, and their physical activity and diet were regular and uniform. Third, the ACB score, which is calculated from the full range of medications that a patient currently uses on a long-term stable basis, provides a good indication of the anticholinergic burden of an individual.

However, some limitations should be considered. First, this work is observational, and causal associations could not be established. Second, serum UA levels measured only once may not adequately reflect the representative concentration levels of the participants over time. Third, the information on antipsychotic medications lacked the detailed blood concentration levels of the medications. Considering plasma concentration as a confounding factor could be good approach. Fourth, the majority of patients did not complete the Positive and Negative Symptom Scales, and the items reflecting negative symptoms were inadequate in BPRS item setting, so negative and positive symptoms were not taken into account more fully. Fifth, the MMSE scale was used to evaluate cognition in this study, while neuropsychological tests are more comprehensive evaluation methods for cognition.

The results supported the hypothesis that serum UA, a blood biomarker, is associated with cognitive status in patients with SCZ during the maintenance period. As a disease-status marker, UA has some prognostic value and could predict cognitive improvement after antipsychotic treatment. However, the potential role of other factors that may clarify the nature of this association warrants further investigation.

## Data availability statement

The raw data supporting the conclusions of this article will be made available by the authors, without undue reservation.

## Ethics statement

The studies involving human participants were reviewed and approved by the Biomedicine Ethics Committee of Baiyun Jingkang Hospital (NFYKDX002). Written informed consent to participate in this study was provided by the participants’ legal guardian/next of kin.

## Author contributions

ZY: conceptualization, methodology, software, formal analysis, and writing—original draft. HL: writing—review and editing. XZ, YHe, SG, DM, and SW: resources. ZH, KW, RZ, QZ, YHu, BC, and HC: data curation. XW: supervision. All authors contributed to the completion of this manuscript.
