# Supplementary material for: Role of uric acid as a biomarker of cognitive function in schizophrenia during maintenance period
Source: Front Psychiatry. 2023 Mar 22;14:1123127. doi: 10.3389/fpsyt.2023.1123127 (PMC10073439; doi:10.3389/fpsyt.2023.1123127)
Supplement: Supplementary file 1 [file Data_Sheet_1.PDF]

Supplementary Material

Table S1: Association between UA and BPRS.

	UA		
	Model 1	Model 2	Model 3
**BPRS**	−0.514 (−1.037, 0.009)	**−0.558 (−1.097, −0.019)**	−0.498 (−1.050, 0.054)
Affect	−0.048 (−0.207, 0.111)	−0.036 (−0.199, 0.128)	−0.011 (−0.179, 0.157)
Positive Symptoms	**−0.222 (−0.414, −0.029)**	**−0.225 (−0.423, −0.026)**	**−0.214 (−0.416, −0.012)**
Negative Symptoms	0.014 (−0.182, 0.211)	−0.033 (−0.235, 0.169)	−0.037 (−0.243, 0.170)
Resistance	−0.157 (−0.337, 0.024)	−0.166 (−0.352, 0.021)	−0.147 (−0.339, 0.045)
Activation	−0.102 (−0.250, 0.047)	−0.099 (−0.252, 0.054)	−0.088 (−0.246, 0.069)

Model 1: Adjusted for age, sex, BMI, course, smoking history, and drinking history.
Model 2: Adjusted for age, sex, BMI, course, smoking history, drinking history, hypertension, diabetes, and dyslipidemia.
Model 3: Adjusted for age, sex, BMI, course, smoking history, drinking history, hypertension, diabetes, dyslipidemia, creatinine, risperidone and aripiprazole.

Abbreviations: UA, uric acid; BMI, body mass index; BPRS, Brief Psychiatric Rating Scale.

Table S2: Statistical description of baseline drugs.

	ACB score	All participants (n = 752)	Low-normal (n = 206)	Middle-normal (n = 206)	High-normal (n = 206)	High (n = 134)	*P* value
Risperidone	1	320 (42.55)	103 (50.00)	70 (33.98)	88 (42.72)	59 (44.03)	**0.012**
Olanzapine	3	264 (35.11)	70 (33.98)	86 (41.75)	69 (33.50)	39 (29.10)	0.091
Sodium valproate	1	176 (3.38)	47 (22.82)	47 (22.82)	44 (21.36)	38 (28.36)	0.495
Clozapine	3	165 (21.94)	50 (24.27)	43 (20.87)	44 (21.36)	28 (20.90)	0.821
Quetiapine	3	91 (12.10)	28 (13.59)	26 (12.62)	26 (12.62)	11 (8.21)	0.486
Perphenazine	3	72 (9.57)	12 (5.83)	21 (10.19)	22 (10.68)	17 (12.69)	0.156
Aripiprazole	1	67 (8.91)	16 (7.77)	18 (8.74)	29 (14.08)	4 (2.99)	**0.005**
Sulpiride	0	66 (8.78)	19 (9.22)	20 (9.71)	14 (6.80)	13 (9.70)	0.682
Alprazolam	1	57 (7.58)	21 (10.19)	14 (6.80)	13 (6.31)	9 (6.72)	0.423
Chlorpromazine	3	47 (6.25)	21 (10.19)	18 (8.74)	15 (7.28)	15 (11.19)	0.580
Haloperidol	1	37 (4.92)	13 (6.31)	11 (5.34)	9 (4.37)	4 (2.99)	0.566
Paliperidone	1	15 (1.99)	7 (3.40)	3 (1.46)	4 (1.94)	1 (0.75)	0.417
Lithium	0	14 (1.99)	3 (1.45)	1 (0.49)	5 (2.43)	5 (3.73)	0.124
Clonazepam	1	12 (1.86)	4 (1.93)	2 (0.97)	5 (2.40)	1 (0.75)	0.621
Diazepam	1	11 (1.46)	3 (1.45)	2 (0.97)	5 (2.43)	1 (0.75)	0.665
Lorazepam	1	10 (1.33)	2 (0.97)	4 (1.94)	3 (1.46)	1 (0.75)	0.856
Sertraline	1	7 (0.93)	2 (0.97)	3 (1.46)	2 (0.97)	0 (0.00)	0.642
Fluoxetine	1	6 (0.80)	3 (1.46)	1 (0.49)	1 (0.49)	1 (0.75)	0.778
Ziprasidone	1	6 (0.80)	0 (0.00)	1 (0.49)	2 (0.97)	3 (2.24)	0.124
Mirtazapine	1	3 (0.40)	1 (0.49)	1 (0.49)	1 (0.49)	0 (0.00)	1.000
Venlafaxine	1	1 (0.13)	1 (0.49)	0 (0.00)	0 (0.00)	0 (0.00)	0.725
Oxazepam	1	1 (0.13)	0 (0.00)	0 (0.00)	1 (0.49)	0 (0.00)	1.000
